# Controlled dye release from a metal–organic framework: a new luminescent sensor for water†

**DOI:** 10.1039/c9ra08753b

**Published:** 2020-01-15

**Authors:** Fan Xiao, Jun Zhang, Jiulin Gan, Ying Tang, Yuanjing Cui, Yang Yu, Guodong Qian

**Affiliations:** State Key Laboratory of Silicon Materials, Cyrus Tang Center for Sensor Materials and Applications, School of Materials Science & Engineering, Zhejiang University Hangzhou 310027 China cuiyj@zju.edu.cn gdqian@zju.edu.cn; State Key Laboratory of Luminescent Materials and Devices, Institute of Optical Communication Materials, South China University of Technology Guangzhou 510640 China

## Abstract

By introducing the dye Rhodamine 6G (R6G) into a metal–organic framework (MOF), Mn-sdc-2 (H_2_sdc = 4,4′-stilbenedicarboxylic acid), with a pore size of 20 × 9.8 Å^2^, the composite R6G@Mn-sdc-2 was obtained. Subsequently, the MOF Mn-sdc-1 with a smaller pore size of 7.5 × 7.5 Å^2^ can be formed through a single-crystal to single-crystal transformation from Mn-sdc-2, thus tightly locking the dye R6G within the pores. Compared with R6G@Mn-sdc-2, R6G@Mn-sdc-1 exhibits a stronger fluorescence emission of R6G. Because the MOF Mn-sdc-1 can reversibly transform back to Mn-sdc-2 in the presence of trace water, the dye R6G can be released. This enables R6G@Mn-sdc-1 to be used as a new luminescent sensor for trace water in organic solvents by monitoring the fluorescence intensity of released R6G. The limit of detection can reach 0.035% in ethanol (v : v), which is among the most sensitive fluorescent water probes.

## Abstract 2

A turn-on trace water sensor was obtained *via* the single-crystal to single-crystal transformation process of a metal–organic framework.

## Graphical abstract

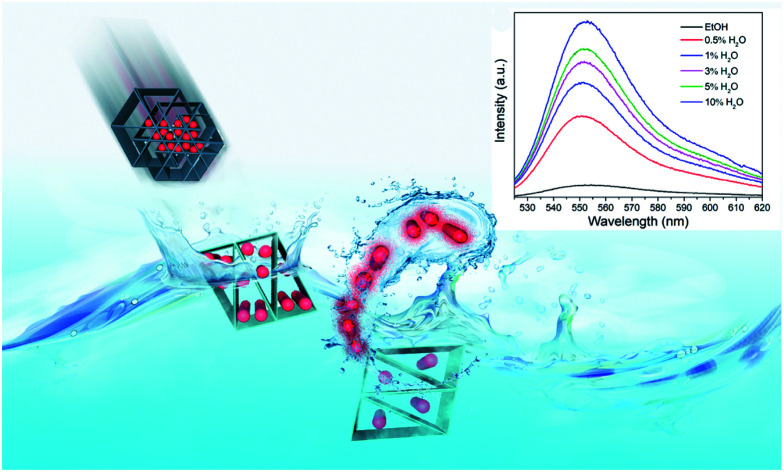

## Introduction

Water plays an indispensable part in human life, however, in the pharmaceutical, fuel, and chemical industries, water content is critical in organic chemical reactions and with moisture-sensitive materials, trace water may be considered as a severe contaminant and an impurity.^1,2^ For instance, bioethanol, a promising green energy source, is rather hygroscopic and soluble in water at any proportion. Therefore, a small amount of water could degrade bioethanol's performance. The criteria for water content in bioethanol is that it must be no more than 0.8% in China and 1.0% in the United States.^3–5^ In lithium ion battery systems, excess water can react with the electrolyte to produce harmful gases, which poses a threat to human life and also impairs the performance of the battery.^6,7^ Thus, it is of high significance to detect trace water in industry. Nevertheless, the traditional solution based on Karl Fischer (K-F) titration methods^8,9^ requires specialized personnel training, high-cost equipment, complicated operation, *etc.* In the meantime, luminescent probes have gained tremendous attention for their handy use, fast response and easy fabrication. A number of luminescent water sensors based on metal–organic frameworks (MOFs) have been reported recently, including ratiometric fluorescence detection,^10–12^ characteristic excited state intramolecular proton transfer (ESIPT) based sensing^13,14^ and so on.

Fluorescent metal–organic frameworks (FL-MOFs) are periodically self-assembled by metal ions or clusters and organic ligands.^15–18^ By ingenious design, the fluorescence centre could be based on metal ions like trivalent lanthanides,^19–21^ guest molecules like introduced dyes or quantum dots,^22,23^ and organic ligands themselves as well.^24^ Besides, given the merits of permanent porosity, it is quite universal for MOFs to exhibit flexibility as the environment changes, the expansion or contraction properties of frameworks may find use in gas-adsorption-separation fields.^25–27^ Furthermore, single-crystal to single-crystal transformation MOFs have been reported recently,^28,29^ the channel topology and size of which can be totally changed under appropriate conditions. This trait is particularly attractive when we introduce guest molecules into the pores and focus on the material properties as the micro-nanostructure varies.

**Fig. 1:**
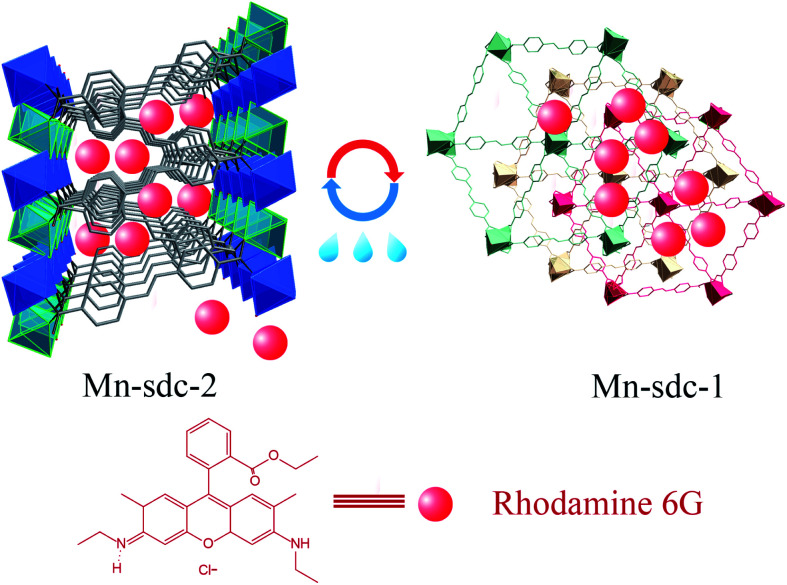
Schematic illustration of the entrapment of the dye Rhodamine 6G into solvent-triggered phase change Mn-MOFs.

Herein, based on solvent-triggered reversible phase change MOFs that have been reported earlier by our group,^30^ we synthesized these two flexible MOFs, namely, Mn-sdc-1 and Mn-sdc-2 (H_2_sdc = 4,4′-stilbenedicarboxylic acid), separately. Mn-sdc-2 has large 1D channels along the *c* axis while Mn-sdc-1 has relatively smaller pores. Therefore, organic dye Rhodamine 6G (R6G) was first introduced into Mn-sdc-2 to form R6G@Mn-sdc-2. Subsequently, by a crystal change process, R6G@Mn-sdc-1 was obtained (Fig. 1). The FL measurements on the two compounds indicate that the fluorescence intensity of R6G in Mn-sdc-1 with smaller pores has been greatly enhanced. Besides, R6G@Mn-sdc-1 could reversibly transform into R6G@Mn-sd-2 in the presence of trace water, at which condition the as-entrapped dye R6G would be released from large pores. Therefore, by monitoring the fluorescence intensity of the released R6G, a turn-on luminescent sensor towards trace water in organic solvent was achieved.

## Results and discussion

### Crystal structure description

**Fig. 2:**
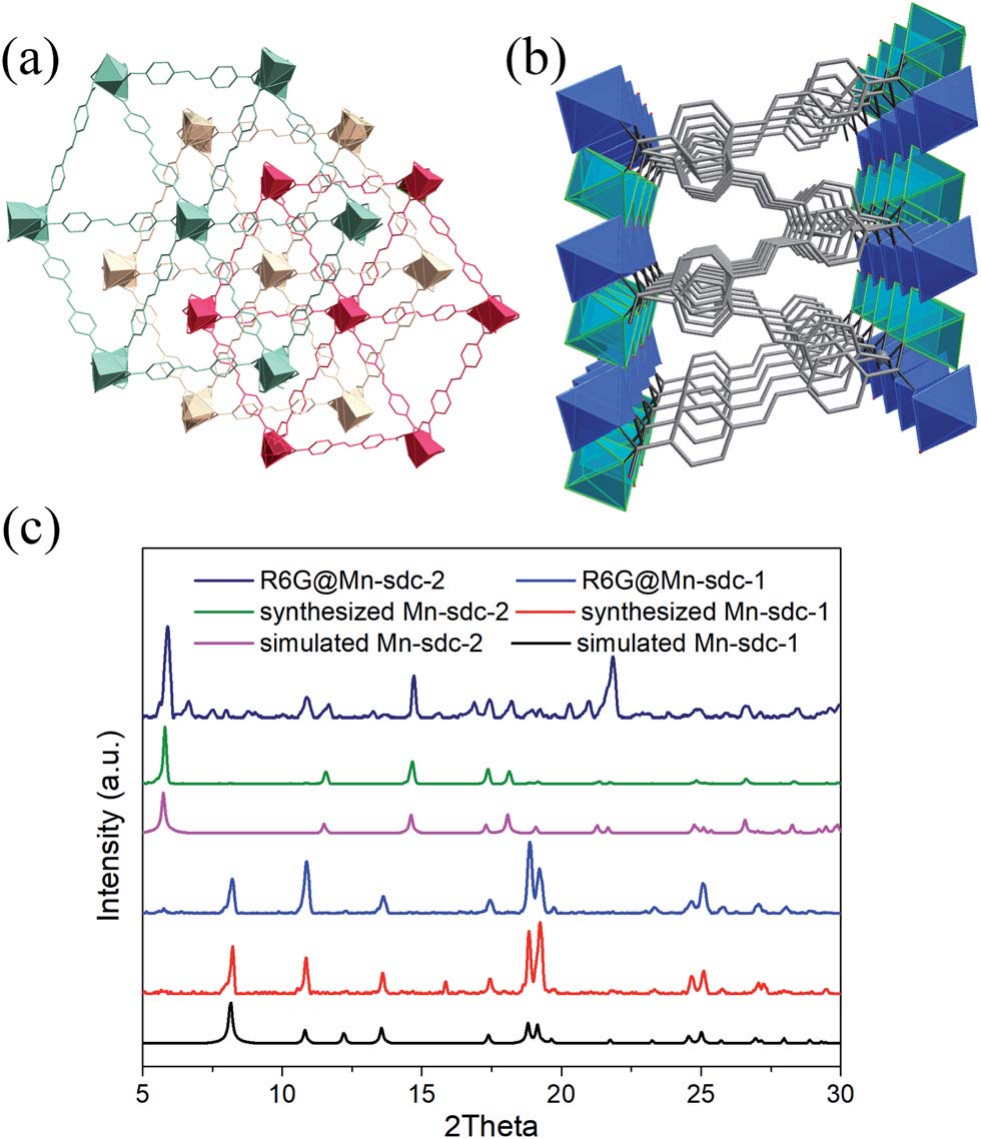
Crystal structure of Mn-sdc-1 (a) and Mn-sdc-2 (b) along the *c* axis. (c) PXRD patterns of the simulated, synthesized and dye encapsulated Mn-MOFs.

The structural and physical nature of the two Mn-MOFs has been reported before,^30^ hence, a brief structural depiction of the MOFs is presented. Mn-sdc-1, formulated as Mn_3_(sdc)_3_(H_2_O)_2_·DMF, belongs to the trigonal space group *R*3̄, each sdc^2−^ ligand coordinates with two trinuclear Mn_3_(RCOO)_6_ secondary building units (SBUs) extending the hexagonal structure to form a two-dimensional layer. The layers further form a close-packed ABC structure^31^ along the *c* axis. Mn-sdc-2, formulated as Mn(sdc)(H_2_O)_2_, crystallizes in the monoclinic system with the *P*2_1_/*c* space group. A Mn^2+^ centre with octahedral geometry is formed by six-coordinated O atoms, these individual Mn^2+^ octahedra are linked by sdc^2−^ to form 2D planes and are further connected by sdc^2−^ ligands, with the planes constituting a 3D framework (Fig. S1†). Now we focus on the pore structure and solvent-triggered phase change properties in this study. As shown in Fig. 1, Mn-sdc-1 exhibits a staggered ABC close-packed topological structure^31^ with a pore size of about 7.5 × 7.5 Å^2^. In contrast, Mn-sdc-2 possesses large 1D channels along the *c* axis, and the pore size is approximately 9.8 × 20 Å^2^. We chose the dye R6G as the guest molecule for two reasons, one is because of its favourable fluorescence properties^32^ and the other is that its molecular size is roughly 16 × 11 Å^2^, which is the intermediate between Mn-sdc-2 and Mn-sdc-1. When the as-synthesized crystals Mn-sdc-2 and Mn-sdc-1 are immersed in a DMF solution of R6G, R6G@Mn-sdc-2 can be obtained at room temperature, but R6G could not be encapsulated into Mn-sdc-1 directly (Fig. S2†) due to its smaller pores. Alternatively, R6G is firstly introduced into large channels of Mn-sdc-2 at room temperature, then, the DMF solution containing R6G@Mn-sdc-2 is transferred to higher temperature to trigger a single-crystal to single-crystal transformation process to obtain R6G@Mn-sdc-1. As shown in Fig. 2, the PXRD patterns of R6G@Mn-sdc-1 and R6G@Mn-sdc-2 are well matched with that of the as-synthesized Mn-MOFs as well as the simulated ones, and the colour of Mn-MOFs changes from light yellow to red clearly demonstrating the successful encapsulation of R6G (Fig. S3†). We carried out FTIR spectroscopic analysis on the dye and MOFs (Fig. S4†). The stretching vibration peak at 1717 cm^−1^ and 1325 cm^−1^ which are attributed to the carboxyl and amine group of the dye R6G, respectively, can be observed in the FTIR spectra of both R6G@Mn-sdc-2 and R6G@Mn-sdc-1. This suggests that the dye R6G has been encapsulated into the MOFs. Besides it is interesting to notice that the transformation temperature for obtaining pure Mn-sdc-2 starts at 100 °C,^30^ but for R6G@Mn-sdc-2, the temperature decreased to 60 °C (Fig. S5†), which indicates that the dye R6G significantly promotes the transformation process. As we have expected, with the heating time increase, the prepared R6G@Mn-sdc-2 in DMF solution gradually transforms to R6G@Mn-sdc-1 (Fig. S6†). Besides the thermogravimetric analysis (Fig. S7†) reveals that dye R6G thermal decomposition occurs at about 200 °C, while R6G@Mn-sdc-1 thermal decomposition temperature increases to 350 °C, this may be due to the MOFs' protective function. Thus R6G@Mn-sdc-1 has an expanded scope of application.

### Fluorescence properties and stability of R6G@Mn-MOF

**Fig. 3:**
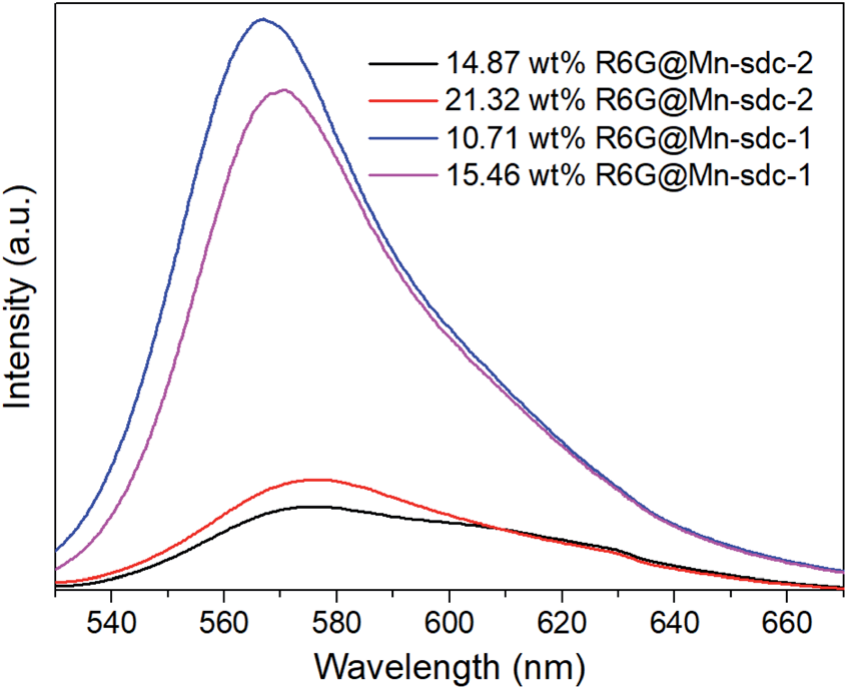
Integrated spectra of R6G@Mn-sdc-2 and R6G@Mn-sdc-1. The excitation wavelength is 515 nm.

**Fig. 4:**
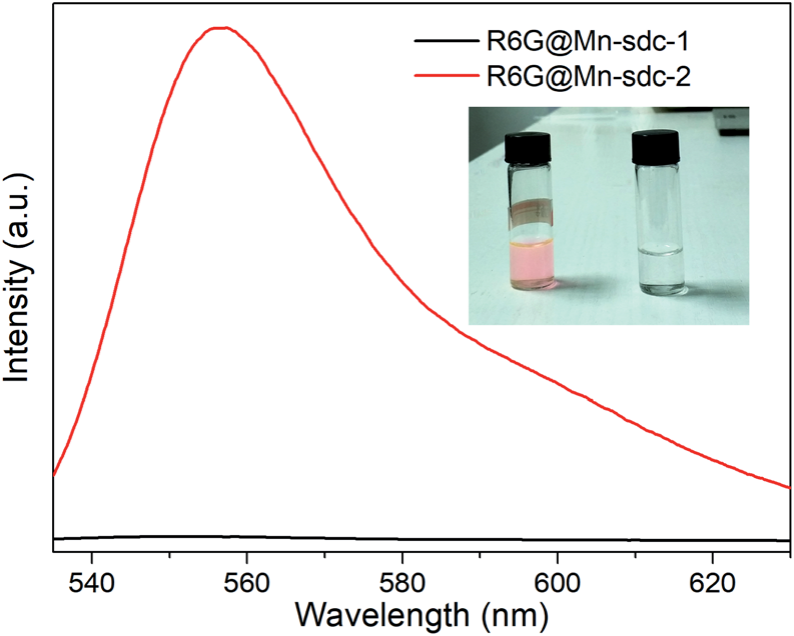
Luminescence spectra of the supernatants after R6G@Mn-sdc-1 and R6G@Mn-sdc-2 immersion for 24 h. Inset: photograph of the supernatant extracted from R6G@Mn-sdc-2 and R6G@Mn-sdc-1. The excitation wavelength is 515 nm.

The as-synthesized crystals Mn-sdc-2 were immersed in a series of DMF solutions of 0.1 mM, 0.5 mM, 1 mM, 1.5 mM and 2 mM R6G concentrations. After being treated at different temperatures, the entrapped dye R6G is calculated to be 0.55 wt%, 5.36 wt%, 14.87 wt%, 21.32 wt% and 26.73 wt% for R6G@Mn-sdc-2, and 0.38 wt%, 3.52 wt%, 10.71 wt%, 15.46 wt% and 20.32 wt% for R6G@Mn-sdc-1. Then the fluorescence properties of R6G@Mn-sdc-2 and R6G@Mn-sdc-1 are investigated at solid state. Upon excitation at 515 nm, the compounds display the broad spectrum of R6G (Fig. S8 and S9†). As the concentration of R6G increases in DMF solution, there exhibits a first increasing and then decreasing trend on the fluorescence intensity of R6G@Mn-sdc-1 and R6G@Mn-sdc-2. When the R6G concentration increased to 2 mM, the spectral peak red-shifts about 20 nm from 555 to 575 nm, which is possibly due to the dye J-aggregate^33,34^ induced dipole moment change of R6G.^35^ Comparing with R6G@Mn-sdc-2, R6G@Mn-sdc-1 exhibits about 4.8 times the fluorescence intensity enhancement at optimal R6G content (Fig. 3). This may be attributed to Mn-sdc-1's close-packed topological structure, which would inhibit R6G from forming dimers^36,37^ and prevent aggregation-caused quenching. Besides, this kind of close-packed small pores in Mn-sdc-1 can serve as a lock to prevent encapsulated R6G from being released. To test this assumption, stability experiments are conducted on R6G@Mn-sdc-1 and R6G@Mn-sdc-2. A certain amount of R6G@Mn-sdc-1 and R6G@Mn-sdc-2 were immersed in high-purity ethanol for 24 h at room temperature, separately. The supernatant of R6G@Mn-sdc-2 solution becomes light red while that of R6G@Mn-sdc-1 remains colourless and transparent (Fig. 4). The fluorescence measurements on the supernatants also demonstrate the considerable difference of the supernatants' R6G content. These results indicate that R6G can be easily released from the large channels of Mn-sdc-2 while being tightly locked in the smaller pores of Mn-sdc-1.

### R6G@Mn-sdc-1 for water sensing

**Fig. 5:**
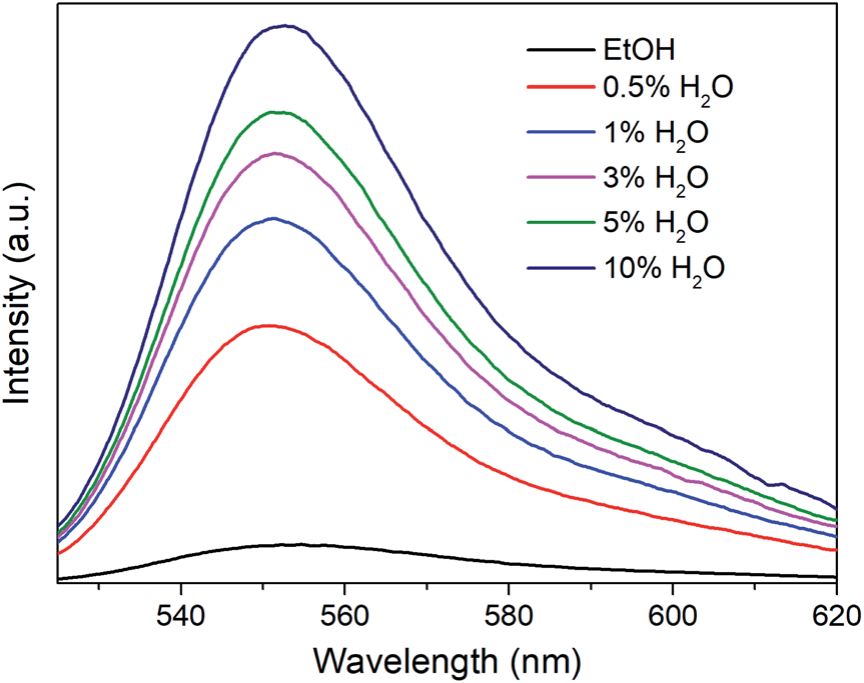
Emission spectra of the supernatant when R6G@Mn-sdc-1 was immersed in ethanol with different water contents. The excitation wavelength is 515 nm.

**Fig. 6:**
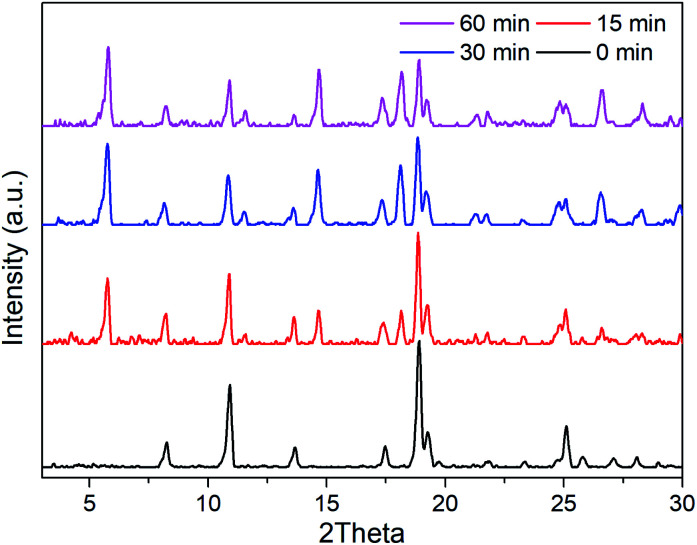
PXRD patterns of R6G@Mn-sdc-1 immersed in ethanol with 5% water content at different times.

R6G can be locked steadily in Mn-sdc-1 and released easily from Mn-sdc-2 and Mn-sdc-1 can transform to Mn-sdc-2 in the existence of trace water in solvent. Based on these two aspects, R6G@Mn-sdc-1 can be considered as a promising fluorescent sensor for trace water in ethanol. We chose the R6G@Mn-sdc-1 prepared in DMF solution containing 1 mM R6G as the samples. Then 2 mg R6G@Mn-sdc-1 was immersed in 2 mL ethanol with water contents varying from 0% to 10% (v : v), after being undisturbed for 5 minutes, the supernatant is extracted and put into a quartz cell for fluorescence study. Upon excitation at 515 nm, the fluorescence spectrum shows the R6G emission peak at around 550 nm. As we have expected, the spectra show a turn-on tendency as the water content increases (Fig. 5). At lower water contents, there is a linear relationship between the fluorescence intensity and water content (Fig. S10†), which can be quantitatively formulated by the Stern–Volmer equation, *I*/*I*_0_ = 1 + *K*_SV_[M],^38^ where *I*_0_ and *I* represent the fluorescence intensity without water and with water, respectively, [M] is the volume content of water in ethanol and *K*_SV_ is the response constant (M^−1^). The Stern–Volmer plot shows a remarkable fluorescence sensitivity toward trace water, the equation is *I*_550_ = 426.65*C*_H_2_O_ + 147.71 (*R*^2^ = 0.9970), where *I*_550_ is the fluorescence intensity at 550 nm. This formula demonstrates an intense turn-on tendency. The limit of detection (LOD) for trace water is computed based on the IUPAC criteria (3*σ*/*K*_SV_),^39^ the LOD value is 0.035%, which is among the most sensitive fluorescence sensors (Table S1†). The sensing mechanism is mainly based on the water-triggered single-crystal to single-crystal transformation, as reported in the previous document.^30^ When it encounters water, Mn-sdc-1 undergoes a lattice rearrangement *via* the rotation of ligand sdc^2−^ and the cleavage and reconstruction of Mn–O bonds to form Mn-sdc-2. Besides, Mn-sdc-1 transforming to Mn-sdc-2 is a spontaneous process which is driven by enthalpy (Δ*E* = −3.02 eV), and water serves as the accelerator for the transformation process. To further confirm the phase transformation mechanism, the cycling test is investigated and the result suggests the potential reusability of the MOF materials (Fig. S11†). In addition, we expand this sensor to other solvents like acetone and acetonitrile, when R6G@Mn-sdc-1 is immersed in acetone or acetonitrile with 1% (v : v) water for a while (Fig. S12 and 13†), the phase transformation and dye releasing phenomena can be monitored, which demonstrates the versatility of this water sensor. We measured the PXRD of R6G@Mn-sdc-1 immersed in ethanol with water (5%) at different times. In the PXRD patterns peaks appear belonging to Mn-sdc-2, which means that Mn-sdc-1 transformed to Mn-sdc-2 immediately (Fig. 6). The results clearly reconfirm the phase transformation mechanism. In addition, originating from the merits of transformable materials, R6G@Mn-sdc-1 can not only detect water at very low concentrations, but also can record the existence of water in some application fields.

## Conclusions

In summary, the organic dye R6G is introduced into large pores of Mn-sdc-2 by the immersion method, then *via* a single-crystal to single-crystal transformation process, the dye R6G is successfully entrapped in small pores of Mn-sdc-1 to obtain R6G@Mn-sdc-1. Compared with R6G@Mn-sdc-2, R6G@Mn-sdc-1 exhibits a stronger fluorescence intensity and prevents R6G from releasing more efficiently. The MOF Mn-sdc-1 can transform to Mn-sdc-2 in the presence of trace water, this property can be applied to detect water, and the detection limit can reach 0.035% in ethanol (v : v), which makes R6G@Mn-sdc-1 a potential fluorescent water sensor in ethanol solution.

## Conflicts of interest

There are no conflicts to declare.

## Supplementary Material

RA-010-C9RA08753B-s001

## References

[cit1] Wang Q., Li X., Wang L., Cheng Y., Xie G. (2005). Ind. Eng. Chem. Res..

[cit2] Bradley D., Williams G., Lawton M. (2010). J. Org. Chem..

[cit3] Abel J., Virtanen S. (2015). Corros. Sci..

[cit4] Lee W.-E., Jin Y.-J., Park L.-S., Kwak G. (2012). Adv. Mater..

[cit5] Li H., Han W., Lv R., Zhai A., Li X.-L., Gu W., Liu X. (2019). Anal. Chem..

[cit6] Ahmed S., Nelson P. A., Dees D. W. (2016). J. Power Sources.

[cit7] Ullman A. M., Jones C. G., Doty F. P., Stavila V., Talin A. A., Allendorf M. D. (2018). ACS Appl. Mater. Interfaces.

[cit8] Karl F. (1935). Angew. Chem..

[cit9] Grunke S. (2001). Food Control.

[cit10] Yu Y., Zhang X. M., Ma J. P., Liu Q. K., Wang P., Dong Y. B. (2014). Chem. Commun..

[cit11] Kim T., Kim Y. (2017). Anal. Chem..

[cit12] Jung H. S., Verwilst P., Kim W. Y., Kim J. S. (2016). Chem. Soc. Rev..

[cit13] Douvali A., Tsipis A. C., Eliseeva S. V., Petoud S., Papaefstathiou G. S., Malliakas C. D., Papadas I., Armatas G. S., Margiolaki I., Kanatzidis M. G., Lazarides T., Manos M. J. (2015). Angew. Chem., Int. Ed..

[cit14] Chen L., Ye J. W., Wang H. P., Pan M., Yin S. Y., Wei Z. W., Zhang L. Y., Wu K., Fan Y. N., Su C. Y. (2017). Nat. Commun..

[cit15] Jiao L., Seow J. Y. R., Skinner W. S., Wang Z. U., Jiang H.-L. (2019). Mater. Today.

[cit16] An H., Li M., Gao J., Zhang Z., Ma S., Chen Y. (2019). Coord. Chem. Rev..

[cit17] Ai J., Chen F.-Y., Gao C.-Y., Tian H.-R., Pan Q.-J., Sun Z.-M. (2018). Inorg. Chem..

[cit18] Xiao J.-D., Jiang H.-L. (2019). Acc. Chem. Res..

[cit19] Yang X., Lin X., Zhao Y., Zhao Y., Yan D. (2017). Angew. Chem., Int. Ed..

[cit20] Pan M., Zhu Y., Wu K., Chen L., Hou Y., Yin S., Wang H., Fan Y., Su C. (2017). Angew. Chem., Int. Ed..

[cit21] Xia T., Zhu F., Jiang K., Cui Y., Yang Y., Qian G. (2017). Dalton Trans..

[cit22] Dong J., Qiao Z., Pan Y., Peh S.-B., Yuan Y.-D., Wang Y., Zhai L., Yuan H., Cheng Y., Liang H., Liu B., Zhao D. (2019). Chem. Mater..

[cit23] Sigalat J. A., Bradshaw D. (2016). Coord. Chem. Rev..

[cit24] Wang B., Lv X.-L., Feng D., Xie L.-H., Zhang J., Li M., Xie Y., Li J.-R., Zhou H.-C. (2016). J. Am. Chem. Soc..

[cit25] Elsaidi S. K., Mohamed M. H., Banerjee D., Thallapally P. K. (2018). Coord. Chem. Rev..

[cit26] Kundu T., Wahiduzzaman M., Shah B. B., Maurin G., Zhao D. (2019). Angew. Chem., Int. Ed..

[cit27] Niu Z., Cui X., Pham T., Lan P.-C., Xing H., Forrest K. A., Wojtas L., Space B., Ma S. (2019). Angew. Chem., Int. Ed..

[cit28] Spirkl S., Grzywa M., Reschke S., Fischer J. K. H., Sippel P., Demeshko S., Nidda H. K., Volkmer D. (2017). Inorg. Chem..

[cit29] Song X.-Z., Song S.-Y., Zhao S.-N., Hao Z.-M., Zhu M., Meng X., Wu L.-L., Zhang H.-J. (2014). Adv. Funct. Mater..

[cit30] Huang Y. K., Zhang J., Yue D., Cui Y. J., Yang Y., Li B., Qian G. D. (2018). Chem.–Eur. J..

[cit31] Kirchon A., Feng L., Drake H. F., Joseph E. A., Zhou H.-C. (2018). Chem. Soc. Rev..

[cit32] Zrimsek A. B., Chiang N., Mattei M., Zaleski S., McAnally M. O., Chapman C. T., Henry A., Schatz G. C., Duyne R. P. (2017). Chem. Rev..

[cit33] Würthner F., Kaiser T. E., Saha-Möller C. R. (2011). Angew. Chem., Int. Ed..

[cit34] Lewkowicz A., Bojarski P., Synak A., Grobelna B., Akopova I., Gryczynski I., Kulak L. (2012). J. Phys. Chem. C.

[cit35] Pourtabrizi M., Shahtahmassebi N., Kompany A., Sharifi S. (2018). J. Fluoresc..

[cit36] Kamino S., Horio Y., Komeda S., Minoura K., Ichikawa H., Horigome J., Tatsumi A., Kaji S., Yamaguchi T., Usami Y., Hirota S., Enomoto S., Fujita Y. (2010). Chem. Commun..

[cit37] Sánchez-Valencia J. R., Toudert J., González-García L., González-Elipe A. R., Barranco A. (2010). Chem. Commun..

[cit38] Liu W., Huang X., Chen C., Xu C., Ma J., Yang L., Wang W., Dou W., Liu W. (2019). Chem.–Eur. J..

[cit39] Ying Y.-M., Tao C.-L., Yu M., Xiong Y., Guo C.-R., Liu X.-G., Zhao Z. (2019). J. Mater. Chem. C.

