# Supplementary material for: Controlled dye release from a metal–organic framework: a new luminescent sensor for water
Source: RSC Adv. 2020 Jan 15;10(5):2722–6. doi: 10.1039/c9ra08753b (PMC9048977; doi:10.1039/c9ra08753b)
Supplement: RA-010-C9RA08753B-s001 [file RA-010-C9RA08753B-s001.pdf]

Electronic Supplementary Material (ESI) for RSC Advances.
This journal is © The Royal Society of Chemistry 2020

Electronic Supporting Information

Controlled Dye Release from Metal-Organic Framework: A New Luminescent Sensor for Water

Fan Xiao,[1] Jun Zhang,[1] Jiulin Gan,[2] Ying Tang,[1] Yuanjing Cui,[1]* Yang Yu,[1] Guodong Qian[1]*

[1]*State Key Laboratory of Silicon Materials, Cyrus Tang Center for Sensor Materials and Applications, School of Materials Science & Engineering, Zhejiang University, Hangzhou 310027, China. E-mail: cuiyj@zju.edu.cn, gdqian@zju.edu.cn*
[2]*State Key Laboratory of Luminescent Materials and Devices and Institute of Optical Communication Materials, South China University of Technology, Guangzhou 510640, China.*

E-mail: cuiyj@zju.edu.cn, gdqian@zju.edu.cn

Materials and measurements

Organic ligand 4-4'-stilbene dicarboxylic acid (H_2sdc) is obtained from Shanghai 3A Chemicals Co. Ltd. Manganese chloride ($MnCl_2$) is purchased from Shanghai 9ding Chemical Biotechnology Co. Ltd. Dye Rhodamine 6G is acquired from Sigma Chemical Co., Ltd. All other reagents and solvents are commercially available and used without further purification.

Powder X-ray diffraction (PXRD) patterns ranging from 3° to 30° are collected on a PANalytical X'Pert Pro X-ray diffractometer using a Cu-Kα (λ=1.542 Å) beam with the recording rate of 5°/min at room temperature.

Fluorescence signals of R6G@Mn-sdc-1, R6G@Mn-sdc-2 and the supernatants are recorded on a Hitachi F4600 fluorescence spectrometer. Xenon lamp serves as an excitation light source. In real experiment, scanning speed was set at 240 nm·min-1, excitation and emission slits width and detector voltage were adjusted to get a proper fluorescence intensity, the test condition remained constant in the whole experiment.

Experimental section

These two MOFs were synthesized according to the literature method[1] with slight modification.
Synthesis of Mn-sdc-1: a mixture of $MnCl_2$ (13 mg, 0.2 mmol), H_2sdc (54 mg, 0.2 mmol), DMF (10 mL) and acetic acid (50 μL) was sealed in a 25 mL Teflon-lined vessel, after sonication for 15 minutes, the homogenous solution was heated at 160 ^{o}C for 48 hours. After cooling slowly down to room temperature, yellow crystals were obtained. The compound was then washed with acetone for three times and dried at 80 ^{o}C for 6 h.
Synthesis of Mn-sdc-2: a mixture of $MnCl_2$ (13 mg, 0.2 mmol), H_2sdc (54 mg, 0.2 mmol), DMF (1 mL) and H_2O (5 mL) was sealed in a 25 mL Teflon-lined vessel, after sonication for 15 minutes, the homogenous solution was heated at 160 ^{o}C for 48 hours. After cooling slowly down to room temperature, needle-like white crystals were obtained. The compound was then washed with acetone for three times and dried at 80 ^{o}C for 6 h.

Synthesis of R6G@Mn-sdc-2: The as-synthesized Mn-sdc-2 crystals were immersed into a series of DMF solution of 0.1 mM, 0.5 mM, 1 mM, 1.5 mM and 2mM dye R6G at room temperature for two days. Subsequently, the resulting crystals were centrifugal separated, washed several times with DMF and acetone until no characteristic color could be observed in the filtrate, then the complexes dried at 80 ^{o}C for 6 h.

Synthesis of R6G@Mn-sdc-1: The as-synthesized crystals Mn-sdc-2 were immersed in a series of DMF solution of 0.1 mM, 0.5 mM, 1 mM, 1.5 mM and 2mM R6G at room temperature for two days to get R6G@MN-sdc-2 first, then the bottles of solution were transferred to 80 ^{o}C for another two days. Subsequently, the resulting crystals were centrifugal separated, washed several times with DMF and acetone until no characteristic color could be observed in the filtrate, then the complexes dried at 80 ^{o}C for 6 h.

Water sensing experiments: A line of bottles containing 2 mL ethanol with different water content were prepared, then 1 mg R6G@Mn-sdc-1 was added into each bottles. After being undisturbed for 5 minutes, the fluorescence measurements were performed on the supernatants to monitor R6G fluorescence intensity.

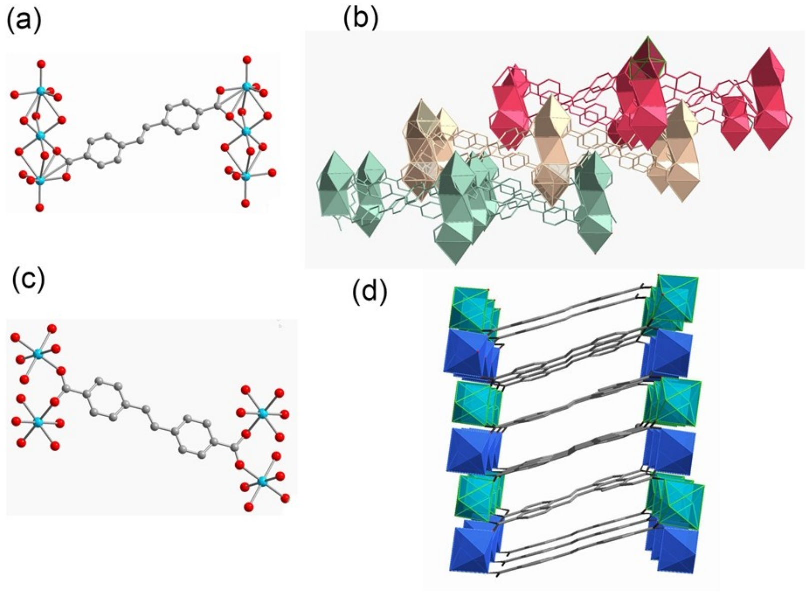

Fig. S1 (a) Coordination environment of Mn^{2+} and sdc^{2-} ligand in Mn-sdc-1; (b) Packing diagram of 1D chains in Mn-sdc-1 along *b*-axis; (c) Coordination environment of Mn^{2+} and sdc^{2-} ligand in Mn-sdc-2; (d) 3D structure of Mn-sdc-2 along *b*-axis.

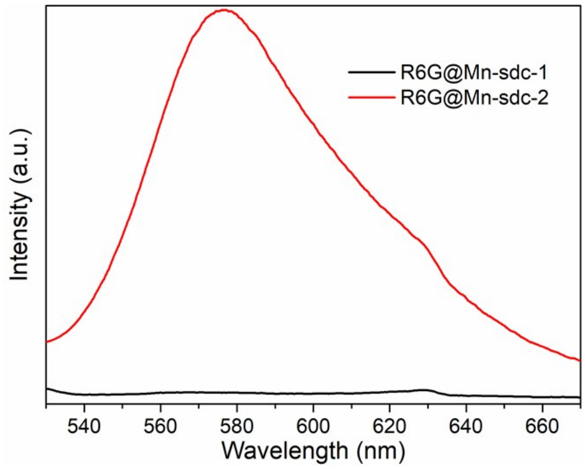

Fig. S2 Luminescence spectra of Mn-MOFs after immersed in R6G solution for 24 h directly. Excited at 515 nm.

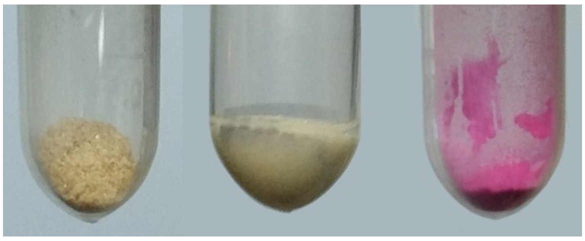

Fig. S3 Photographs of Mn-sdc-1, Mn-sdc-2 and R6G@Mn-sdc-2, respectively.

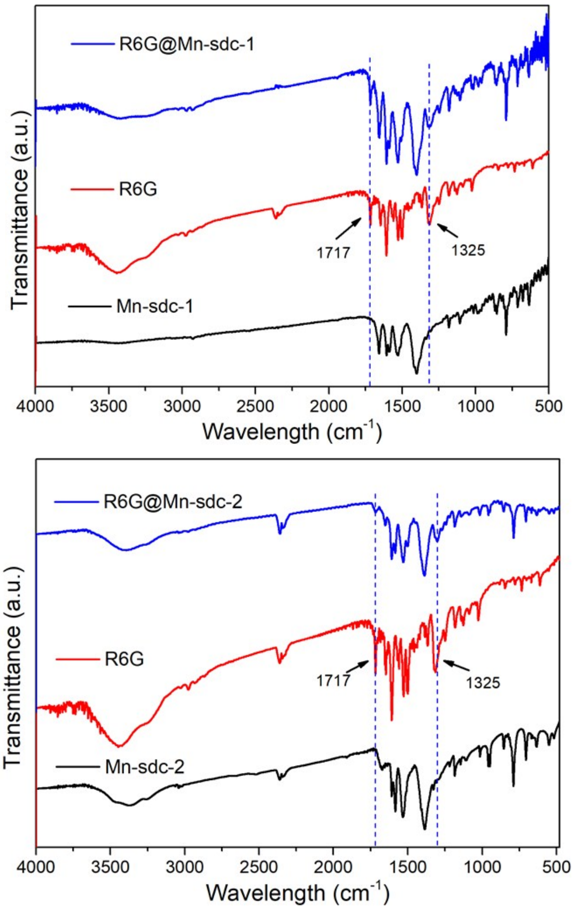

Fig. S4 Fourier transform infrared spectra of dye R6G, Mn-MOFs and R6G@Mn-MOFs.

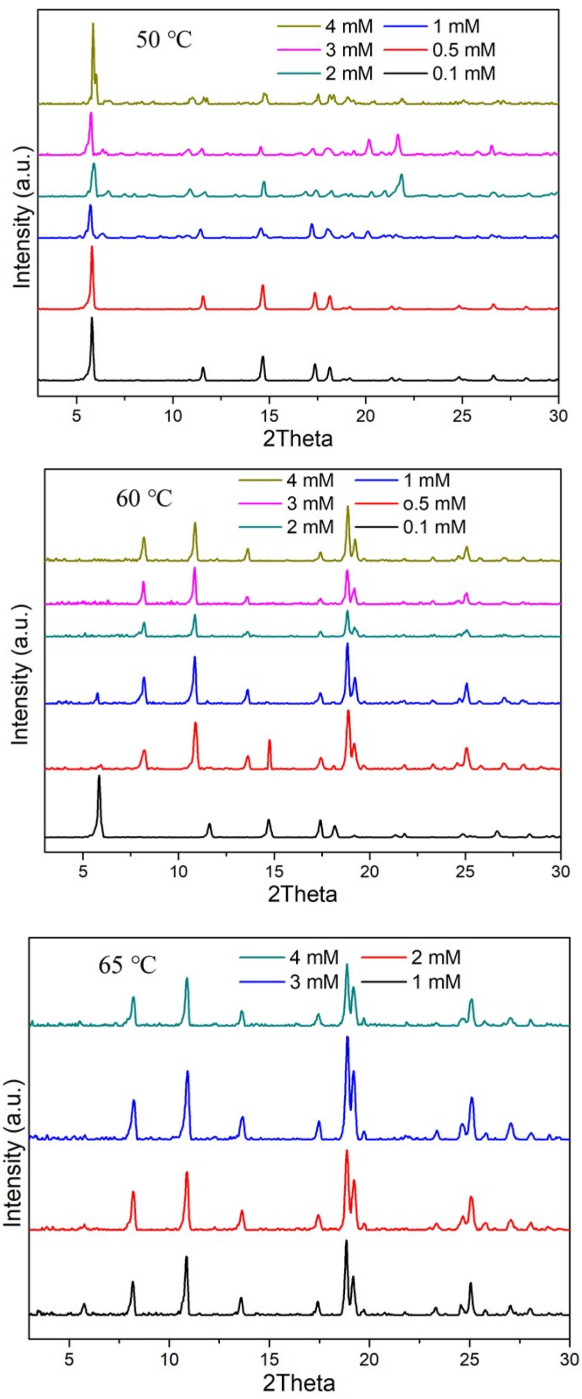

Fig. S5 PXRD patterns of Mn-sdc-2 immersed in different R6G concentrations and at different temperatures.

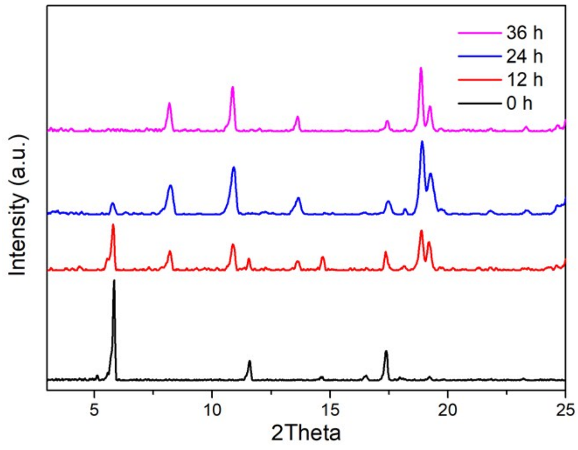

Fig. S6 PXRD patterns of R6G@Mn-sdc-2 immersed in 1mM R6G concentrations at 80 ^{o}C for different time.

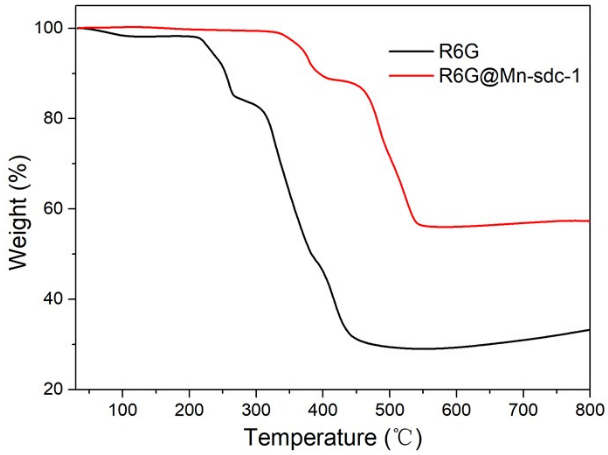

Fig. S7 TGA curves of dye R6G and R6G@Mn-sdc-1.

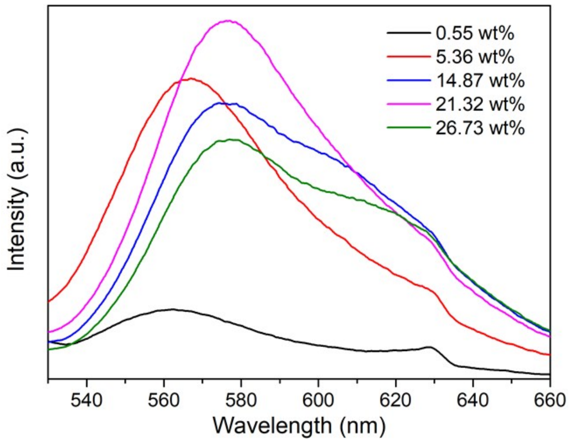

Fig. S8 Luminescence spectra of different R6G@Mn-sdc-2, excited at 515 nm.

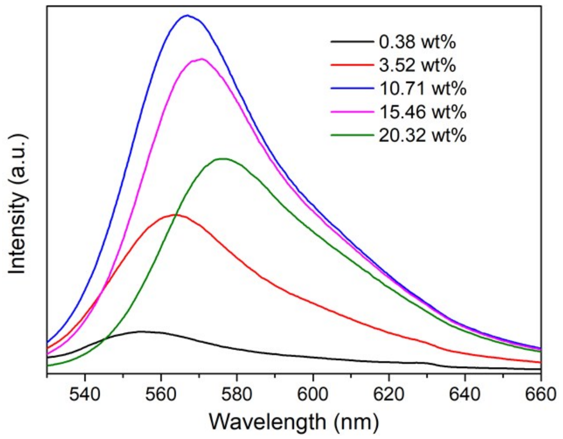

Fig. S9 Luminescence spectra of different R6G@Mn-sdc-1, excited at 515 nm.

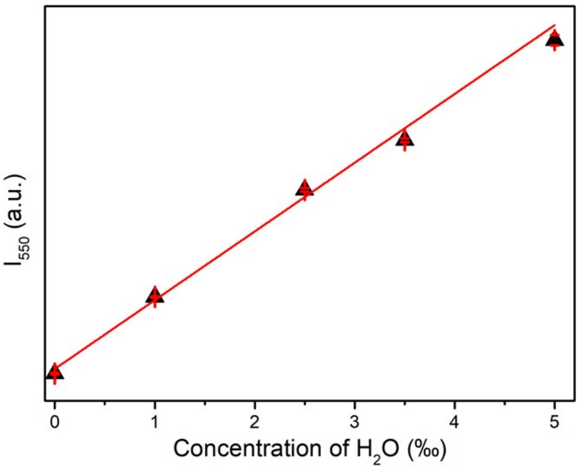

Fig. S10 The linear relationship between the fluorescence intensity and water content.

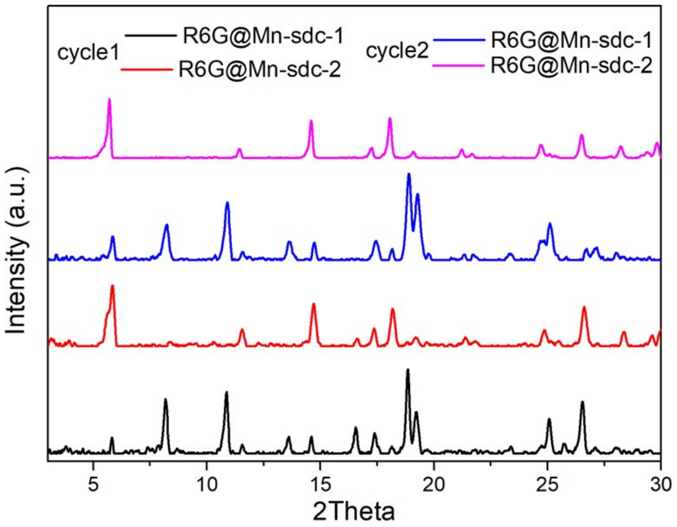

Fig. S11 The PXRD patterns of R6G@Mn-sdc-1 and R6G@Mn-sdc-2 cyclic transformation.

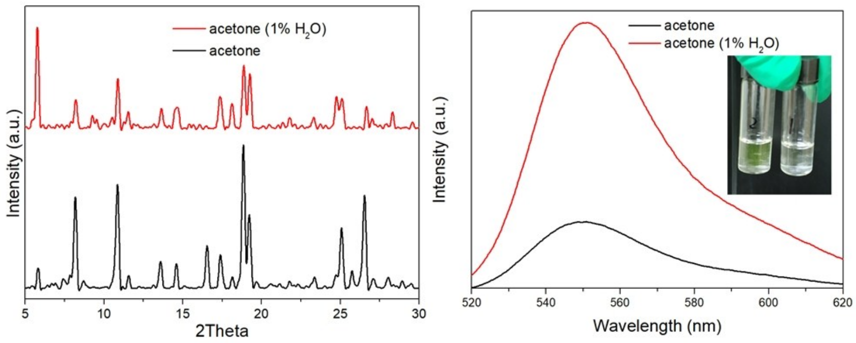

Fig. S12 The PXRD pattern of R6G@Mn-sdc-1 immersed in acetone with or without trace water; and the R6G fluorescence in supernatant.

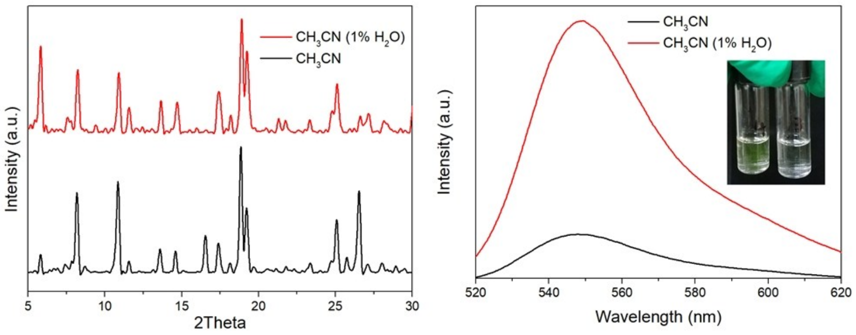

Fig. S13 The PXRD pattern of R6G@Mn-sdc-1 immersed in acetonitrile with or without trace water; and the R6G fluorescence in supernatant.

.

Table. S1 different MOFs sensors for trace water sensing

materials	LOD	Sensing type	Reference
**Eu$_{0.02}$Dy$_{0.18}$-MOF**	0.1%	Ratiometric fluorescence	2
**Ru@MIL-NH$_2$**	0.02%	Ratiometric fluorescence	3
**AEMOF-1'**	<0.05%	Turn-on fluorescence	4
**Zn-MOF**	<0.05%	Switchable luminescence	5
**QG-loaded MOF**	0.015%	Turn-on fluorescence	6
**Polymer/HKUST-1**	<0.1%	Colorimetric fluorescence	7
**Mn-MOFs**	0.035%	Turn-on fluorescence	This work

Reference

1 Y. K. Huang, J. Zhang, D. Yue, Y. J. Cui, Y. Yang, B. Li and G. D. Qian, Chem. Eur. J., 2018, 24, 13231.
2 H. Li, W. Han, R. Lv, A. Zhai, X. –L. Li, W. Gu and X. Liu, *Anal. Chem.*, 2019, **91**, 2148.
3 H. –Q. Yin, J. –C. Yang and X. –B. Yin, *Anal. Chem.*, 2017, **89**, 13434.
4 A. Douvali, A. C. Tsipis, S. V. Eliseeva, S. Petoud, G. S. Papaefstathiou, C. D. Malliakas, I. Papadas, G. S. Armatas, I. Margiolaki, M. G. Kanatzidis, T. Lazarides and M. J. Manos, *Angew. Chem., Int. Ed.*, 2015, **2013**, 1651.
5 L. Chen, J. W. Ye, H. P. Wang, M. Pan, S. Y. Yin, Z. W. Wei, L. Y. Zhang, K. Wu, Y. N. Fan and C. Y. Su, *Nat. Commun.*, 2017, **8**, 1.
6 Y. Cai, L. Feng, Y. Hua, H. Liu, M. Yin, X. Lv, S. Li and H. Wang, *Chem. Commun.*, 2018, **54**, 13595.
7 A. M. Ullman, C. G. Jones, F. P. Doty, V. Stavila, A. A. Talin and M. D. Allendorf, *ACS Appl. Mater. Interfaces*, 2018, **10**, 24201.
